# Delayed Onset of Age-Dependent Changes in Ultrastructure of Myocardial Mitochondria as One of the Neotenic Features in Naked Mole Rats (*Heterocephalus glaber*)

**DOI:** 10.3390/ijms20030566

**Published:** 2019-01-29

**Authors:** Lora Bakeeva, Valeria Vays, Irina Vangeli, Chupalav Eldarov, Susanne Holtze, Thomas Hildebrandt, Vladimir Skulachev

**Affiliations:** 1 A.N. Belozersky research institute of Physico-Chemical Biology, Lomonosov Moscow State University, 119991 Moscow, Russia; bakeeva@belozersky.msu.ru (L.B.); valeriya.vays@yandex.ru (V.V.); sim870@mail.ru (I.V.); chupalav@protonmail.ch (C.E.); 2 Department of Reproduction Management, Leibniz-Institute for Zoo and Wildlife Research, Alfred-Kowalke-Str. 17, 10315 Berlin, Germany; holtze@izw-berlin.de (S.H.); hildebrandt@izw-berlin.de (T.H.)

**Keywords:** aging, naked mole rat, neoteny, mitochondria, ultrastructure, cardiomyocytes, electron microscopy

## Abstract

In this study, the ultrastructure of mitochondria in cardiomyocytes of naked mole rats (*Heterocephalus glaber*) aged from 6 months to 11 years was examined. Mitochondria in cardiomyocytes of naked mole rats have a specific ultrastructure that is different from those in cardiomyocytes of other mammalian species studied to date. In contrast to mitochondria of other mammalian cardiomyocytes, where the internal space is completely filled by tightly packed parallel rows of cristae, mitochondria in cardiomyocytes of naked mole rats have a chaotic pattern of cristae organization with wave-like contours. Gradual formation of mitochondrial ultrastructure occurs in naked mole rats for many years. Two mitochondrial populations are developed to the age of 5 years. In addition to the main population, there are some large organelles which exceed normal sizes by two to three times. Most cristae in these mitochondria are assembled into small groups, which form the curved and ring-like structures. The appearance of some specific structural changes (i.e. bundles of parallel cristae) is observed in the mitochondrial population of naked mole rat after 11 years of age. However, these bundles are very rare and of sporadic nature. Morphometric analysis has shown that the superficial density of the inner mitochondrial membrane is similar in all examined age groups of naked mole rats: 21.1 at 6 months; 23.21 at 3 years; 23.55 at 5 years; and 20.8 at 11 years. This level is almost two times lower than in other animals studied (mice and rats). The data demonstrate that pathological changes in mitochondrial apparatus are not present in naked mole rats at least until the age of 11 years. The mitochondrial apparatus corresponds to the phenotype in young animals, thus being another neotenic feature in naked mole rats.

## 1. Introduction

Currently, it is hypothesized that the longevity of some animal species is due to retarded aging and the prolongation of juvenility (neoteny) [1]. The term “neoteny” was proposed by D. Kollman in 1905 [2]; later it was used to describe the “persistence of juvenile features at a mature age” [3]. Neoteny is considered one of possible mechanisms underlying the longevity of a highly social mammal species, the naked mole rat (*Heterocephalus glaber*). The term neoteny was initially applied to naked mole rat by R. Alexander in 1991 [4]. The naked mole rat is a small rodent weighing up to 35 g inhabiting underground labyrinths in arid and semi-arid areas of Kenya, Ethiopia, and Somalia. Naked mole rat colonies have a complex organization. A queen and one to three high-rank males are the colony leaders; all other colony members are workers not involved in reproduction. Their longevity is one of the most interesting features of naked mole rats. The maximum lifespan for naked mole rats in captivity is more than 31 years. They very rarely suffer from diseases, such as cancer or diabetes, and cardiovascular and neurological disorders are very rare or completely absent among the causes of mortality in these mammals. At present, much attention is paid to the detection of neotenic features in adult naked mole rats. A detailed list of all neotenic features in these animals has not yet been provided. To date, our group has provided information about 43 neotenic features in naked mole rats [4]. They predominantly include physiological and biochemical characteristics, whereas no descriptions of ultrastructures of various tissues and cell organelles of naked mole rats are yet published in the literature. 

According to current views, brain, skeletal muscle, and myocardium are the tissues most susceptible to age-dependent deterioration. Therefore, the ultrastructural studies of these tissues in naked mole rats are highly significant. It is well-known that mitochondria are the most important organelles regulating cell metabolism; mitochondria are one of the first structures reacting to various stimuli and environmental factors. They possess high morphological variability depending on functional activity of the cell. Mitochondria are the organelles which undergo the most pronounced structural changes at aging. At present, no studies of mitochondria ultrastructure in naked mole rat are available. We examined the ultrastructural features of mitochondria in cardiomyocytes of naked mole rats of various age groups, from newborns to 11 years of age, both in queens and subordinates. No differences in electron microscopic images were found between these two castes. This study continues our preliminary researches [5]. Here, we describe the results of our research into four age groups: 6 months, 3 years, 5 years, and 11 years.

## 2. Results and Discussion

Our studies demonstrated that mitochondria in cardiomyocytes of naked mole rats have a remarkable and unique ultrastructure different from those in cardiomyocyte mitochondria in all animal species examined to date. 

**Figure 1:**
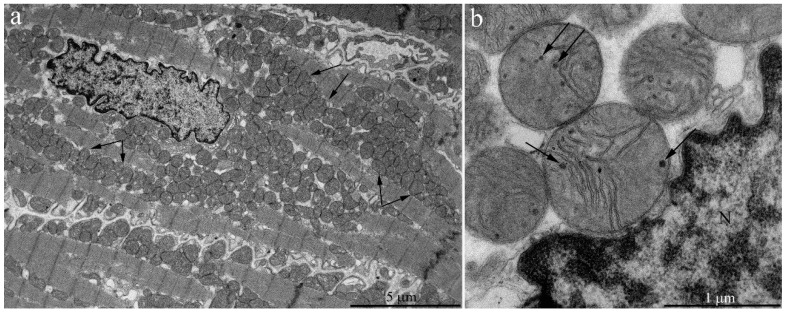
Electron microscopic images of the ultrastructure of a left ventricular cardiomyocyte from a 6-month-old naked mole rat: (**a**) General view photo at low magnification. Long rows of mitochondria along myofibrils are visible. Arrows show mitochondria located within a sarcomere. (**b**) Ultrastructure of a single mitochondrion at higher magnification. Arrows show the electron-dense granules in the matrix. N—cardiomyocyte nucleus.

**Figure 2:**
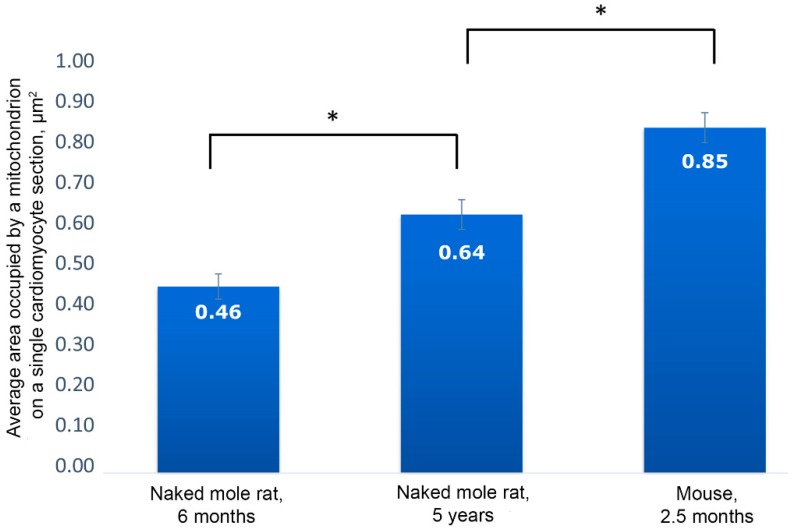
Results of morphometric analysis of cardiomyocyte mitochondrial pool. The average area occupied by a mitochondrion on a single cardiomyocyte section in naked mole rats aged 6 months and 5 years, and in mice aged 2.5 months; * *p* < 0.05.

Figure 1a shows the general ultrastructure of a left ventricle cardiomyocyte in a naked mole rat aged 6 months. Long rows of numerous mitochondria can be seen; mitochondria are mostly oval-shaped and located along the myofibrils. Mitochondria are very small so that several mitochondria are present in a single sarcomere (Figure 1a). At the same time, mitochondria in cardiomyocytes of mammals widely used in experimental biology (including mice and rats) are predominantly (after 1 month of age and older) present as elongated structures located within one or several sarcomeres each, according to the commonly accepted classic description of myocardial ultrastructure [6]. Morphometric analysis showed that the cross-section area of a single mitochondrion in a 2.5-month-old mouse is 0.85 μm^2^ compared to 0.46 μm^2^ in a naked mole rat, i.e. almost two times smaller (Figure 2).

**Figure 3:**
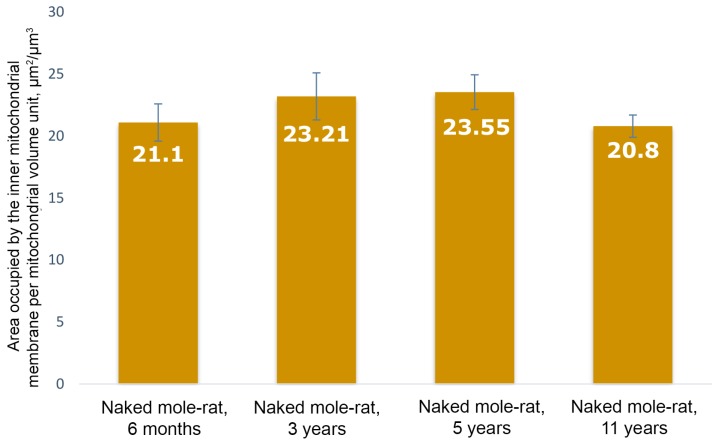
Results of morphometric analysis of cardiomyocyte mitochondria ultrastructure in naked mole rats. The area of inner membrane per mitochondrial volume unit in naked mole rats aged 6 months, 3 years, 5 years, and 11 years.

The internal structure of mitochondria in naked mole rats is also unusual (Figure 1b). In contrast to mitochondria in cardiomyocytes of other mammals, where inner mitochondrial space is completely filled by tightly packed parallel rows of cristae [5,7], mitochondria in cardiomyocytes of naked mole rats have a chaotic pattern of cristae distribution with wave-like contours. The mitochondrial matrix is well-developed and contains some dense granules (shown by arrows in Figure 1b). The surface density of the inner mitochondrial membrane in a 6-month naked mole rat is 21.1 μm^2^/μm^3^ (Figure 3). As we have shown previously [7] in 3-month old rats with mitochondria exhibiting the internal ultrastructure typical for mammal myocardium, the surface density of the inner mitochondrial membrane was 41.3 μm^2^/μm^3^, which is almost two times bigger compared to naked mole rat. 

**Figure 4:**
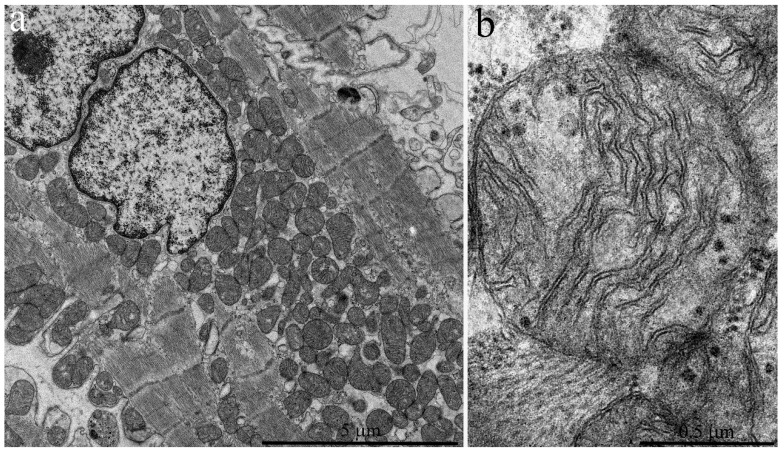
Electron microscopic images of the ultrastructure of a left ventricular cardiomyocyte from a three-year-old naked mole rat: (**a**) General view at low magnification. Mitochondrial aggregates in perinuclear area are observed. (**b**) Ultrastructure of a single mitochondrion at higher magnification.

Cardiomyocytes of three-year old naked mole rats (Figure 4a,b) may contain enlarged mitochondria both near the nucleus and between myofibrils. The internal structure of mitochondria is modified with more ordered arrangement of cristae. We found no ultrastructural changes in cardiomyocyte mitochondria in contrast to literature data on experimental animals (rats and mice) aged 2.5–3 years [8,9,10]. The surface density of the inner mitochondrial membrane in three-year old naked mole rats exhibits no significant change relative to six-month old animals; it is equal to 23.21 μm^2^/μm^3^ (Figure 3).

**Figure 5:**
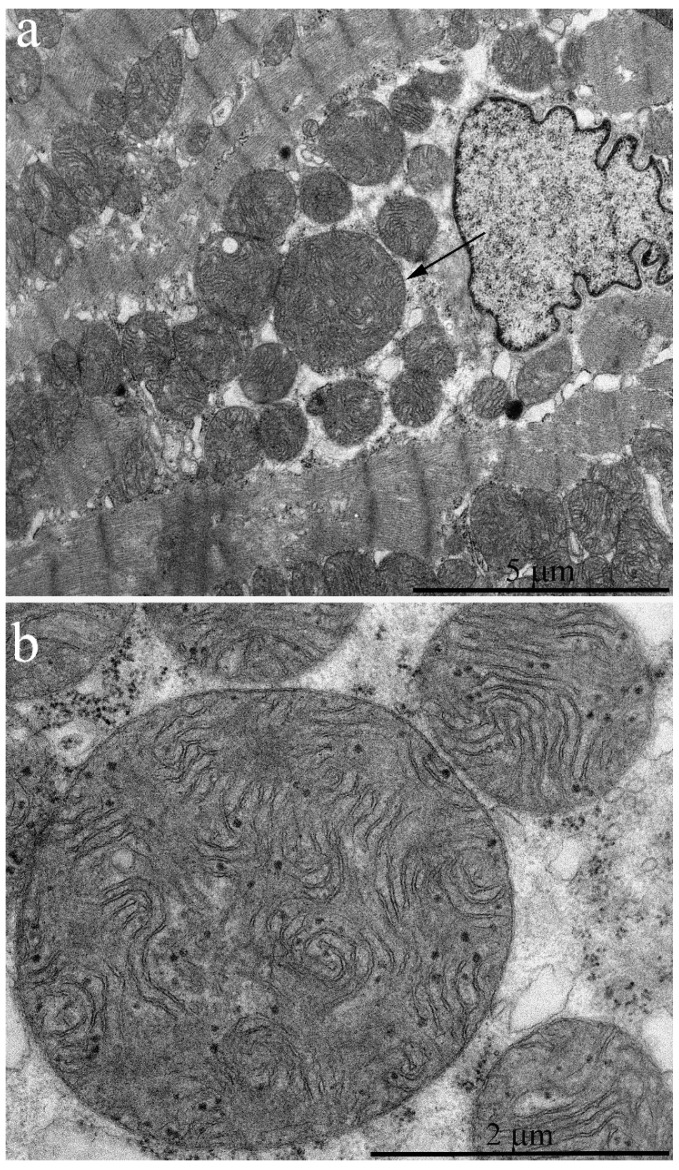
Electron microscopic image of the ultrastructure of a left ventricular cardiomyocyte from a 5-year-old naked mole rat: (**a**) General view at low magnification. The arrow indicates the large mitochondrion shown at higher magnification in (**b**), which shows the ultrastructural features of the large mitochondrion.

Cardiomyocytes of the five-year-old naked mole rats (Figure 5a,b) contain a population of very large mitochondria, exceeding normal sizes by two to three times (shown by arrow in Figure 5a). Figure 5b shows the same mitochondrion at greater magnification. Usually, only a single mitochondrion of such ultrastructure is located in the perinuclear area. Groups of several mitochondria of this ultrastructure may be located between myofibrils. Not only the size but also inner structure of such mitochondria is quite unusual. Figure 5b shows that most cristae are joined into small groups forming curved and ring-like structures, which uniformly fill the entire mitochondrial inner space. The mitochondrial matrix also contains electron-dense granules. 

The main population of mitochondria demonstrates a further increase in size up to five years of age. The cross-section area of a single mitochondrion from a naked mole rat is increased up to 0.64 μm^2^ at five years of age (Figure 2). The inner ultrastructure of the main mitochondrial population demonstrates the more ordered arrangement of wave-like cristae and well-developed matrix containing granules (shown by the arrow in Figure 5b). However, despite a more pronounced wave-like structure of cristae, the surface density of the inner mitochondrial membrane stays the same: 23.55 μm^2^/μm^3^ (Figure 3). As we have demonstrated previously [7], the significant decrease in surface density of the inner mitochondrial membrane is one of the morphometric parameters that characterizes the myocardial mitochondria ultrastructure in mice and rats of two years of age and older (this parameter was twofold less in two-year-old rats). Thus, we found no ultrastructural signs characteristic of aging despite the age of five years.

It is well known that mitochondrial ultrastructure formation in mice and rats is completed already by three months of age, and typical destructive changes in cell organelles—first of all in mitochondria—develop and progress after the age of 18 months [8]. We observed certain destructive changes both in general structure of mitochondrial apparatus in individual cardiomyocytes and in the ultrastructure of individual organelles of naked mole rat cardiomyocytes only since 11 years of age. 

**Figure 6:**
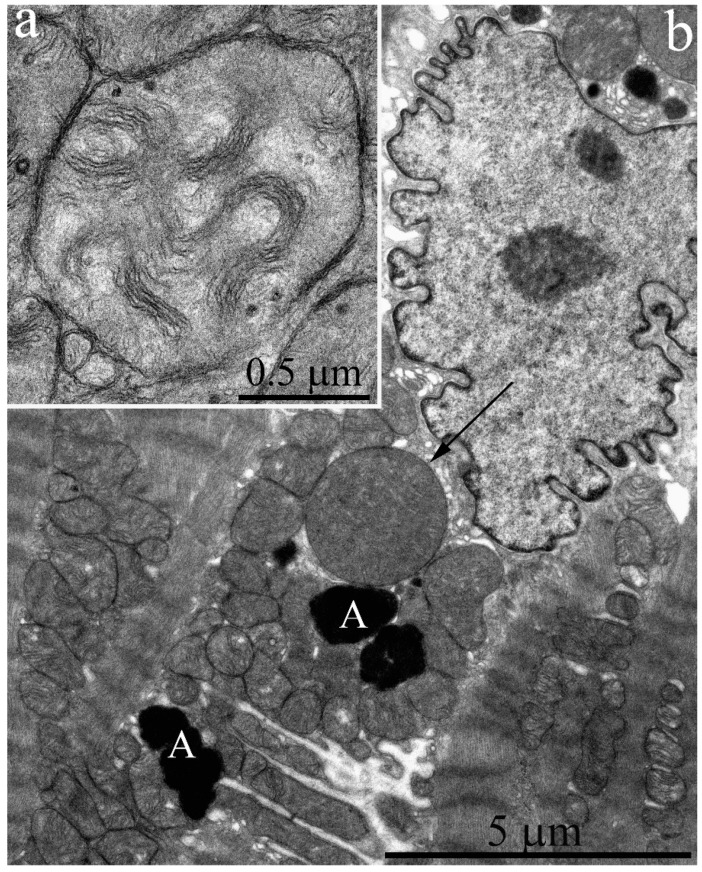
Electron microscopic image of the ultrastructure of a left ventricular cardiomyocyte from an 11-year-old naked mole rat. (**a**) Ultrastructure of mitochondria from the main population. (**b**) General view at low magnification. The arrow indicates a large mitochondrion, which is shown at greater magnification in Figure 7. A—autophagosome.

**Figure 7:**
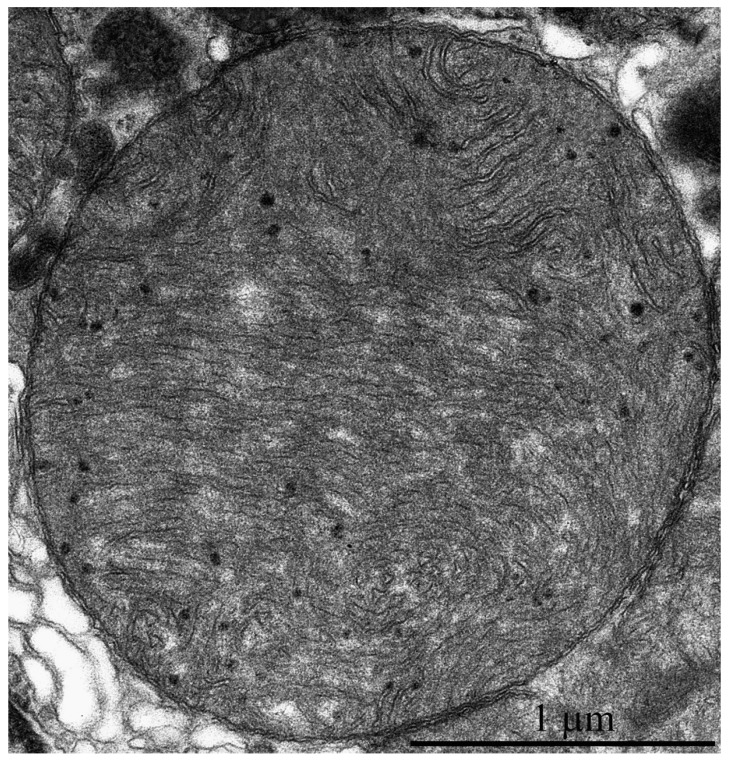
Electron microscopic image of the ultrastructural features of a large mitochondrion from a left ventricular cardiomyocyte from 11-year-old naked mole rat.

Figure 6b shows a fragment of cardiomyocyte from the left ventricle of a naked mole rat aged 11 years. Similar to cardiomyocytes of five-year-old naked mole rats, the perinuclear area contains only a single large mitochondria with unusual ultrastructure. However, mitochondrial ultrastructure is partially impaired in this population, both in perinuclear and inter-fibrillary mitochondria. Figure 7 shows the ultrastructure of a mitochondrion, indicated by the arrow in Figure 6b. It is seen that the elements of ultrastructure organization typical for this mitochondrial population are preserved only in the peripheral area of the organelle. The middle area of the mitochondrion contains tightly packed and folded membranes, where matrix and intermembrane space are hard to distinguish. This local degradation of mitochondrial ultrastructure was observed in megamitochondria of cardiomyocytes in the biopsy from a 58-year-old human male [11]. 

The main mitochondrial population ultrastructure in naked mole rat cardiomyocytes is also changed after 11 years. Figure 6a shows that mitochondrial cristae are arranged into separate twisted stacks. The main inner mitochondrial space is filled with matrix. It should be noted that mitochondrial matrix is electron-transparent. Despite visually observed changes in the mitochondrial inner ultrastructure, the surface density of the inner mitochondrial membrane remains the same (20.8 μm^2^/μm^3^). It is almost two times lower than the level in adult mice and rats [7]. This finding is in line with the results by Holtze et al. [5] that show the adenine nucleotide content in myocardial mitochondria of naked mole rats is twice lower than in adult mice and closer to that in murine embryo. These results demonstrate the absence of age-related abnormalities in the main mitochondrial population of naked mole rats up to 11 years of age. 

**Figure 8:**
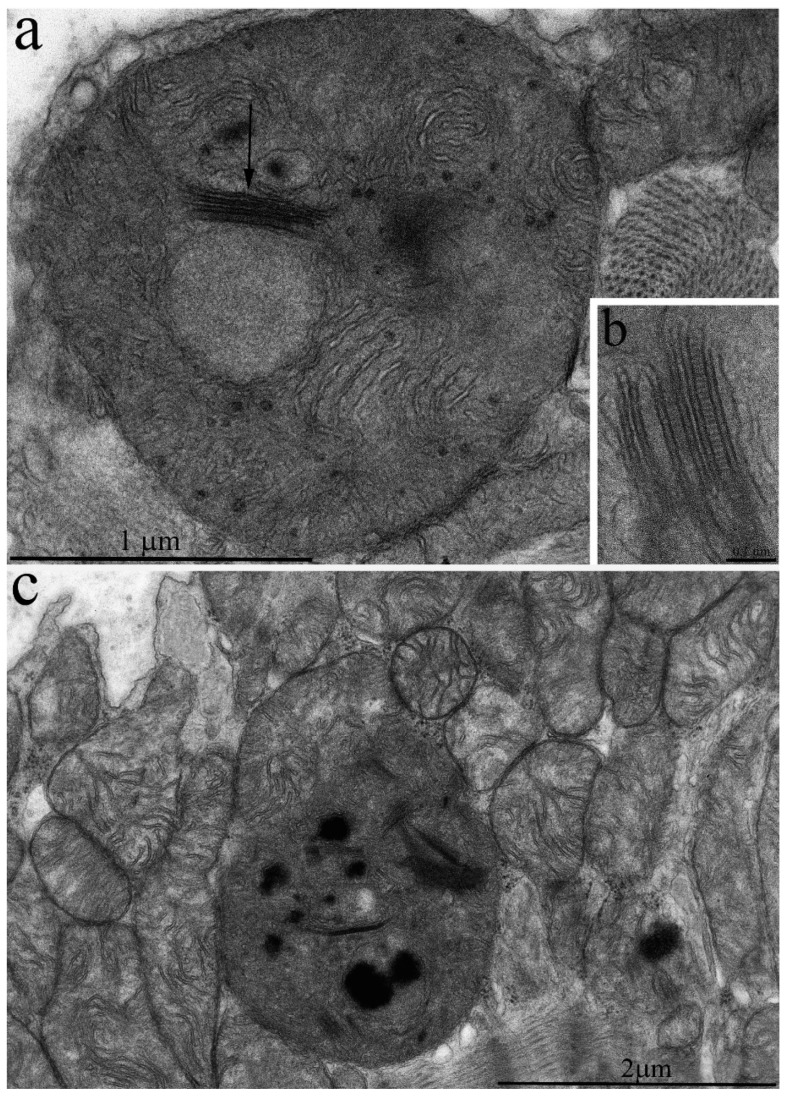
Ultrastructure of individual cardiomyocyte mitochondria in a 11-year-old naked mole rat: (**a)** Mitochondria with disrupted crista ultrastructure. The arrow shows the appearance of a bundle of cristae. (**b**) The bundle at higher magnification. (**c**) Mitochondrial population of a left ventricular cardiomyocyte. Mitochondria with bundles are very rare.

**Figure 9:**
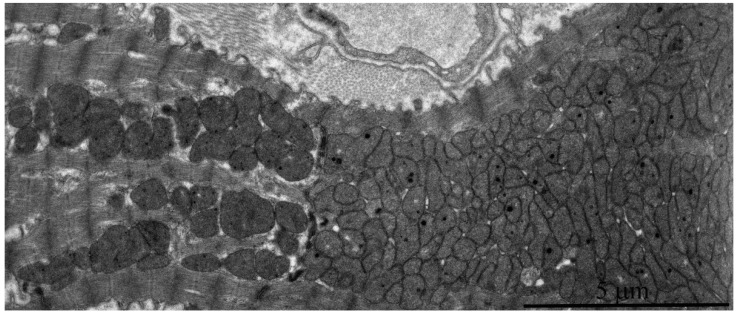
Electron microscopic image of the ultrastructure of two adjacent left ventricular cardiomyocytes from an 11-year-old naked mole rat. It is visible that the left cardiomyocyte has a normal ultrastructure of mitochondrial apparatus while the right cardiomyocyte contains huge numbers of small mitochondria.

However, it should be noted that isolated mitochondria with disrupted crista ultrastructure are present in very rare cases (Figure 8c), and transverse intermembrane junctions are formed in the inter-membrane space (Figure 8a–c). In the literature, they are most often named “membrane junctions” or “intra-crystal junctions”. A vast number of authors have observed such intra-mitochondrial structures [12,13,14,15]. They are usually considered a sign of mitochondrial aging [15,16]. In addition, some local alterations also develop in the general mitochondrial apparatus organization of 11–year-old naked mole rats. Figure 9 shows two adjacent cardiomyocytes: the left one has a normal mitochondrial ultrastructure, while the right cardiomyocyte demonstrates a huge mass of chaotically arranged, multiple, and rather small mitochondria of variable morphology. The literature considers this pattern of mitochondrial ultrastructure as a sign of mitochondrial proliferation observed in rats aged 26–27 months. It is associated with the functional transformation of mitochondrial apparatus [8]. 

The gradual formation of mitochondrial ultrastructure in naked mole rat cardiomyocytes over many years, and the appearance of initial destructive changes of mitochondria only from 11 years of age, as found in our studies, are possibly one of the factors making naked mole rats highly resistant to cardiovascular disorders. Thus, the detailed necropsy examinations of several hundred naked mole rat carcasses showed no cases of cardiac pathology [17]. 

Our data correlate well with the findings of Penz et al. [18], who discovered a pronounced delay in brain development in naked mole rats. The authors showed prolonged postnatal morphogenesis of hippocampal neurons that remained uncompleted up to the age of 8–10 years in naked mole rats. 

However, currently there are no studies of mitochondrial ultrastructure in naked mole rats. Our results entirely contradict the literature data on age-related changes of mitochondrial ultrastructure in rat and mouse myocardium [7,8,9]. The established view states age-associated progression of degenerative processes: these alterations are prominent both in rats and in mice in the age range of 2–2.5 years, and degeneration affects the majority of the mitochondrial population. Our observations of mitochondrial ultrastructure and the morphometric analysis of the obtained data demonstrate the development of strong mitochondrial apparatus during the first five years in naked mole rats and no signs of degenerative processes at the ultrastructural level are observed up to 11 years. The ultrastructure of the main cardiomyocyte mitochondrial population in 11-year-old naked mole rats and morphometric analysis findings (the similar surface densities of inner mitochondrial membranes in naked mole rats aged 11 years, 6 months, and 5 years) demonstrate that 11-year-old naked mole rats do not yet develop the any wide-spread pathological alterations in mitochondrial apparatus. Another sign of neoteny is the ultrastructural similarity of the mitochondrial apparatus in 11-year-old naked mole rats to the phenotype of young animals. Therefore, the results of our studies support the hypothesis about neoteny in adult naked mole rats, with preservation of juvenile characteristics in the tissue structure and function during their elongated ontogenesis [1,18].

## 3. Materials and Methods

### 3.1. Animals

#### 3.1.1. Naked Mole Rats

Four groups of naked mole rats (aged 6 months, 3 years, 5 years, and 11 years) and two groups of mice (aged 2, 5 and 30 months) were studied. Each group contained four animals. Naked mole rat colonies were kept at the Leibniz-Institute for Zoo and Wildlife Research (Berlin, Germany) in artificial plexiglass labyrinths. The temperature in the system was maintained at 26–29 °C, and relative humidity was rather high, 60–80%. The boxes contained wooden litter, small twigs, and pieces of paper. Fresh food was available daily without restrictions and included sweet potatoes, carrots, apples, fennel, groats with vitamins and minerals, and oat flakes. Experiments were approved by the Ethics Committee of Landesamt für Gesundheit und Soziales, Berlin, Germany (#ZH 156; G 0221/12; T 0073/15).

#### 3.1.2. Mice

Two groups of animals of (aged 2.5 and 30 months) were used. Each group contained four animals. The animals were housed with natural illumination, an air temperature of 22 ± 2 °C, and free access to water and feed (RK-120-1; Laboratorsnab, Moscow, Russia). All procedures were performed according to the European Union Council Directive 86/609/EEC.

### 3.2. Electron Microscopy

For this examination, tissue of the left ventricular wall was excised and fixed with 3% glutaraldehyde solution (Sigma-Aldrich, St. Lois, MO, USA) in 0.1 M phosphate buffer (pH 7.4) for 2 h at 4 °C. Furthermore, it was fixed with 1% osmium tetroxide for 1.5 h and then dehydrated in alcohol series with increasing alcohol concentrations of 50%, 60%, 70%, 80%, and 96% (70% alcohol contained 1.4% uranyl acetate; Serva, Heidelberg, Germany) to enhance contrast. After that, samples were embedded in Epon812 epoxy resin. A series of ultrathin sections was prepared with an ultramicrotome (Leica, Wetzlar, Germany) and stained with lead. The obtained preparations were imaged and photographed under a JEM1400 electron microscope (JEOL, Akishima, Tokyo, Japan), operating at the accelerating voltage of 100 kV and beam current of 65 μA, equipped with a QUEMESA camera (Olympus, Shinjuku, Tokyo, Japan) and processed with the software provided with the electron microscope (EMSIS GmbH, Muenster, Germany).

### 3.3. Morphometry and Statistical Analysis

For morphometric examination, 40 electron microscopy images at higher magnification (×15,000) and 20 images at lower magnification (×1500) of myocardial tissue from each group were selected and processed by Adobe^©^ Photoshop^®^ graphical software suite v.13.0.1 (“Adobe”, San Jose, CA, USA). The surface density of the inner membrane—area of the inner mitochondrial membrane (µm^2^) per unit of volume of mitochondria (µm^3^)—was calculated using high-magnification images by a previously described method [6]. Low-magnification images were used to calculate the total area occupied by mitochondria on a single cardiomyocyte section and the total count of mitochondria per section. Using this data and Photoshop embedded measurement tools, we obtained the mean area occupied by a single mitochondrion on a cardiomyocyte section. Statistical processing of the morphometric data was performed by STATISTICA 8 software suite (StatSoft Inc., Thulsa, OK, USA). Significance level was checked by the Mann—Whitney test with Bonferroni correction, if necessary.
